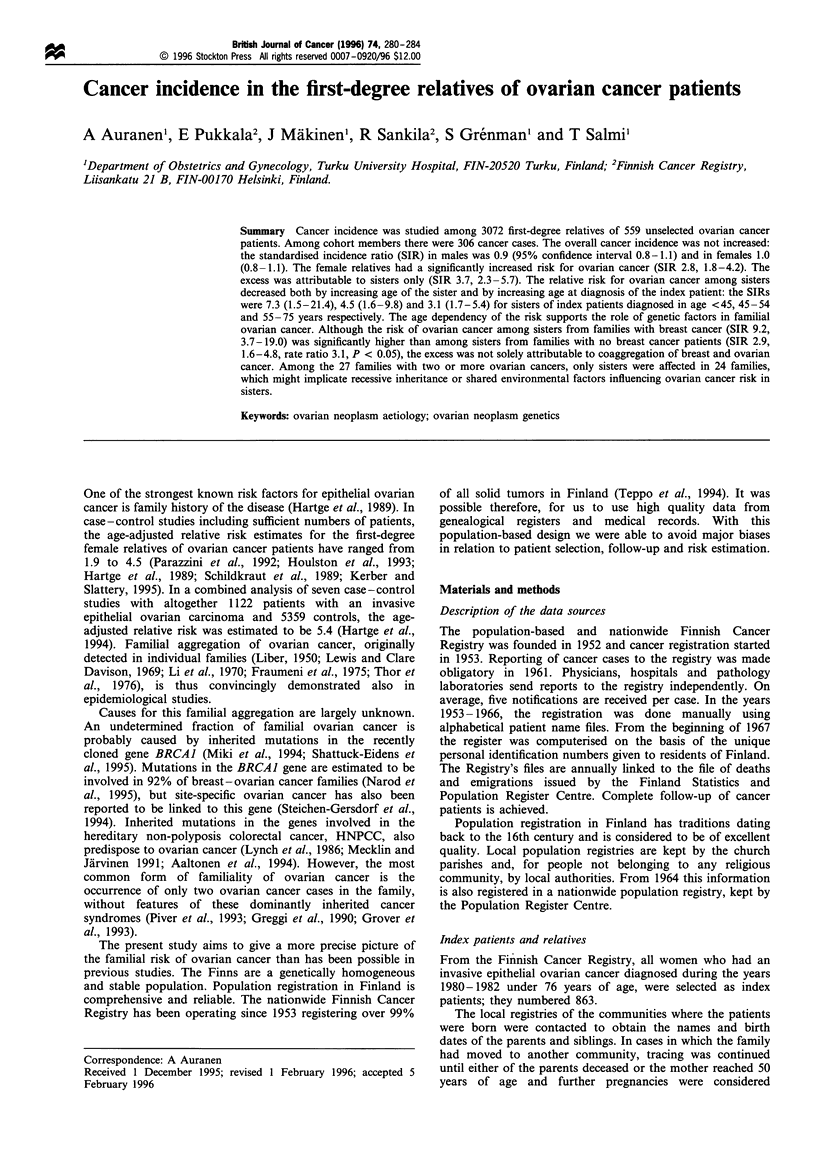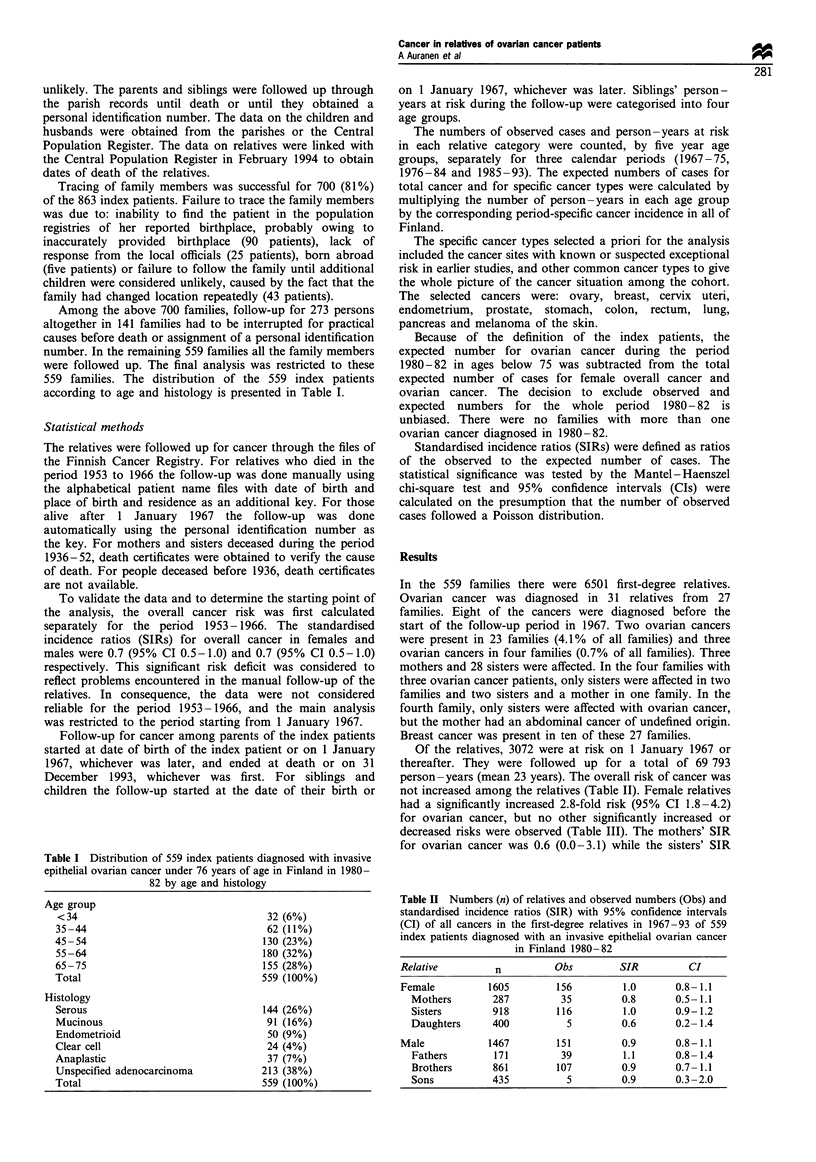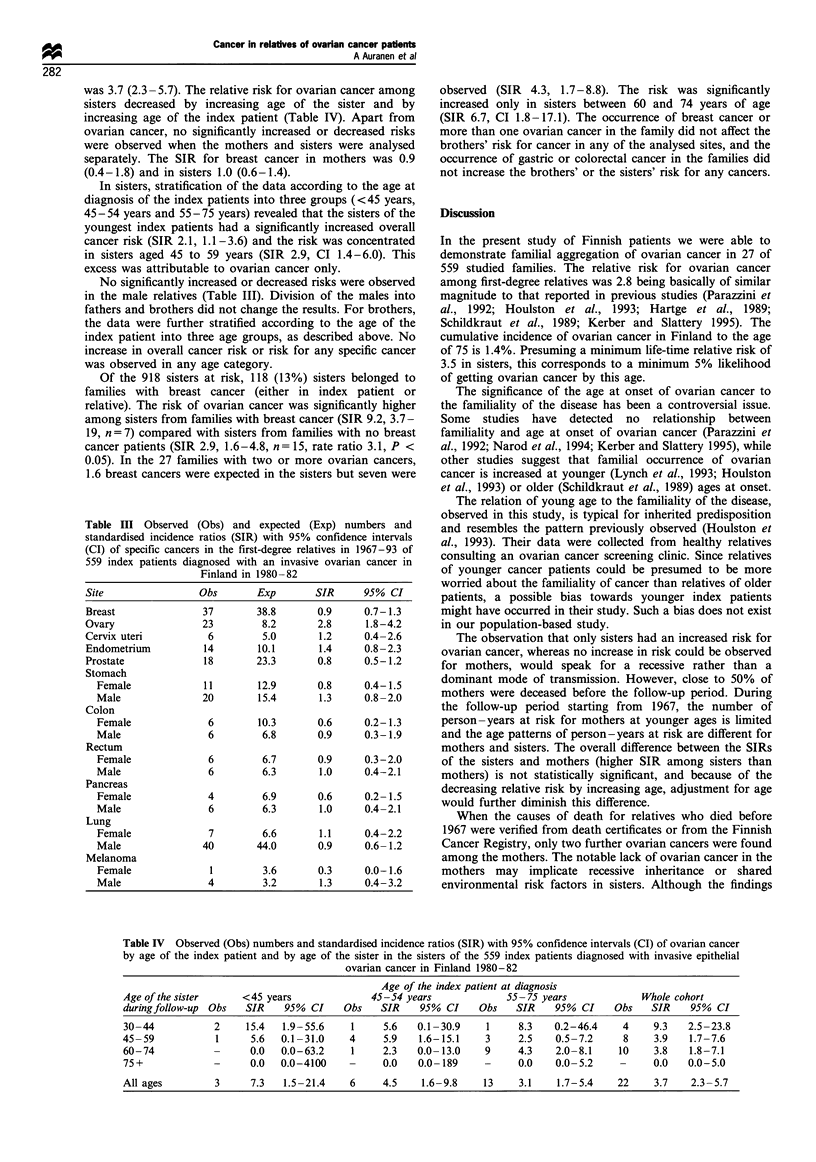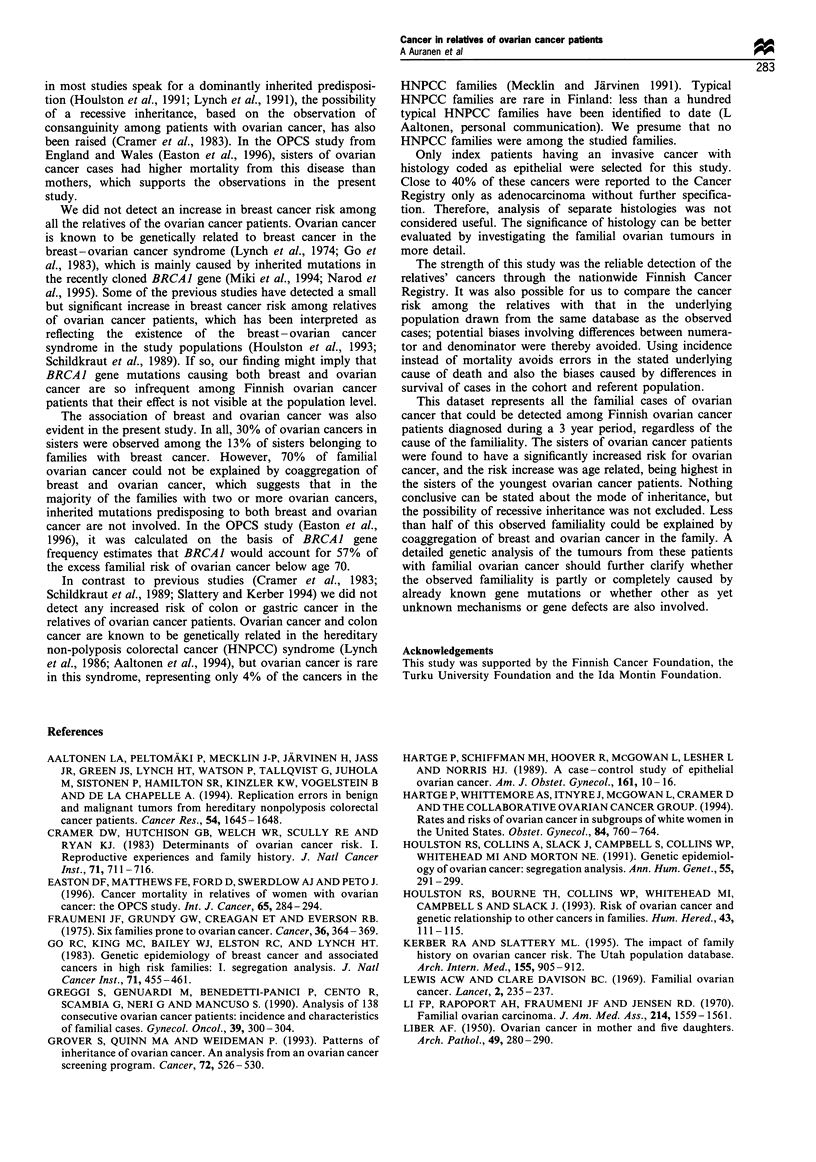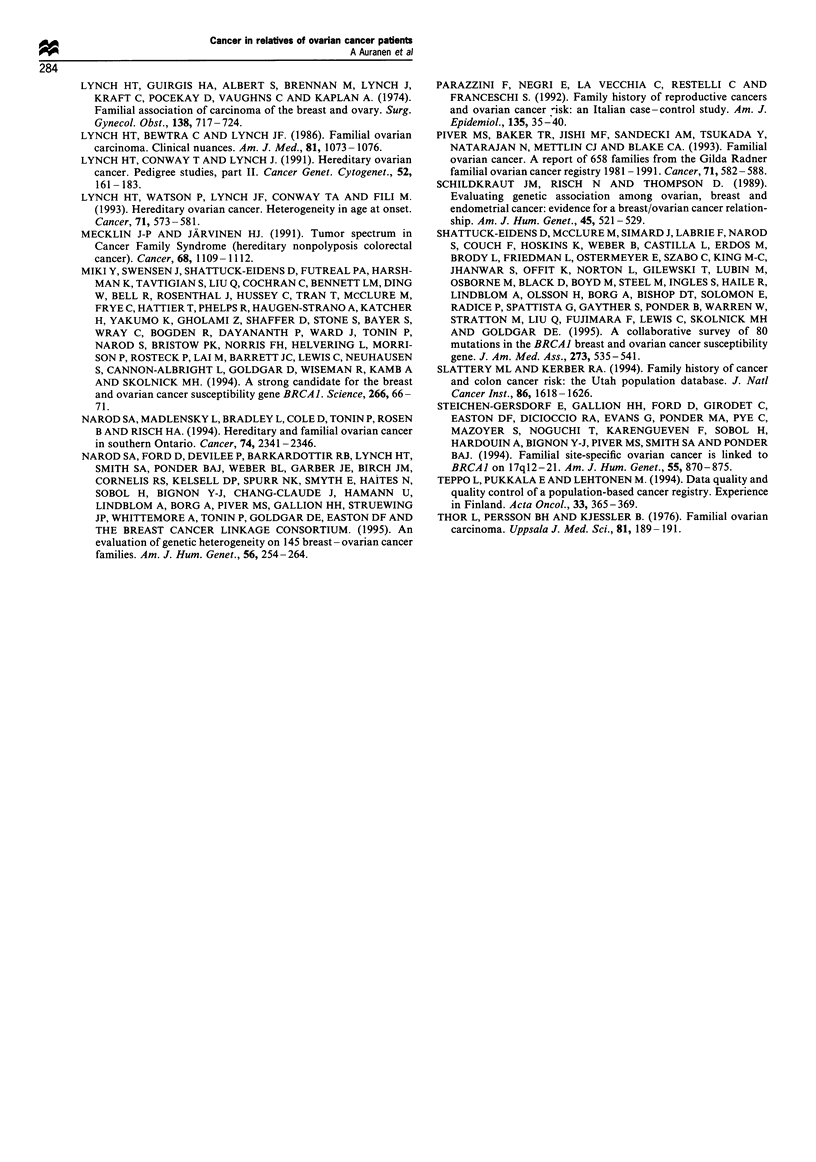# Cancer incidence in the first-degree relatives of ovarian cancer patients.

**DOI:** 10.1038/bjc.1996.352

**Published:** 1996-07

**Authors:** A. Auranen, E. Pukkala, J. Mäkinen, R. Sankila, S. Grénman, T. Salmi

**Affiliations:** Department of Obstetrics and Gynecology, Turku University Hospital, Finland.

## Abstract

Cancer incidence was studied among 3072 first-degree relatives of 559 unselected ovarian cancer patients. Among cohort members there were 306 cancer cases. The overall cancer incidence was not increased: the standardised incidence ratio (SIR) in males was 0.9 (95% confidence interval 0.8-1.1) and in females 1.0 (0.8-1.1). The female relatives had a significantly increased risk for ovarian cancer (SIR 2.8, 1.8-4.2). The excess was attributable to sisters only (SIR 3.7, 2.3-5.7). The relative risk for ovarian cancer among sisters decreased both by increasing age of the sister and by increasing age at diagnosis of the index patient: the SIRs were 7.3 (1.5-21.4), 4.5 (1.6-9.8) and 3.1 (1.7-5.4) for sisters of index patients diagnosed in age < 45, 45-54 and 55-75 years respectively. The age dependency of the risk supports the role of genetic factors in familial ovarian cancer. Although the risk of ovarian cancer among sisters from families with breast cancer (SIR 9.2, 3.7-19.0) was significantly higher than among sisters from families with no breast cancer patients (SIR 2.9, 1.6-4.8, rate ratio 3.1, P < 0.05), the excess was not solely attributable to coaggregation of breast and ovarian cancer. Among the 27 families with two or more ovarian cancers, only sisters were affected in 24 families, which might implicate recessive inheritance or shared environmental factors influencing ovarian cancer risk in sisters.


British Journal of Cancer (1996) 74, 280-284
© 1996 Stockton Press All rights reserved 0007-0920/96 $12.00

### Cancer incidence in the first-degree relatives of ovarian cancer patients

A Auranen[1], E Pukkala[2], J Mäkinen[1], R Sankila[2], S Grénman[1] and T Salmi[1]

[1]*Department of Obstetrics and Gynecology, Turku University Hospital, FIN-20520 Turku, Finland;* [2]*Finnish Cancer Registry, Liisankatu 21 B, FIN-00170 Helsinki, Finland.*

**Summary** Cancer incidence was studied among 3072 first-degree relatives of 559 unselected ovarian cancer patients. Among cohort members there were 306 cancer cases. The overall cancer incidence was not increased: the standardised incidence ratio (SIR) in males was 0.9 (95% confidence interval 0.8-1.1) and in females 1.0 (0.8-1.1). The female relatives had a significantly increased risk for ovarian cancer (SIR 2.8, 1.8-4.2). The excess was attributable to sisters only (SIR 3.7, 2.3-5.7). The relative risk for ovarian cancer among sisters decreased both by increasing age of the sister and by increasing age at diagnosis of the index patient: the SIRs were 7.3 (1.5-21.4), 4.5 (1.6-9.8) and 3.1 (1.7-5.4) for sisters of index patients diagnosed in age <45, 45-54 and 55-75 years respectively. The age dependency of the risk supports the role of genetic factors in familial ovarian cancer. Although the risk of ovarian cancer among sisters from families with breast cancer (SIR 9.2, 3.7-19.0) was significantly higher than among sisters from families with no breast cancer patients (SIR 2.9, 1.6-4.8, rate ratio 3.1, $P < 0.05$), the excess was not solely attributable to coaggregation of breast and ovarian cancer. Among the 27 families with two or more ovarian cancers, only sisters were affected in 24 families, which might implicate recessive inheritance or shared environmental factors influencing ovarian cancer risk in sisters.

**Keywords:** ovarian neoplasm aetiology; ovarian neoplasm genetics

One of the strongest known risk factors for epithelial ovarian cancer is family history of the disease (Hartge *et al.*, 1989). In case-control studies including sufficient numbers of patients, the age-adjusted relative risk estimates for the first-degree female relatives of ovarian cancer patients have ranged from 1.9 to 4.5 (Parazzini *et al.*, 1992; Houlston *et al.*, 1993; Hartge *et al.*, 1989; Schildkraut *et al.*, 1989; Kerber and Slattery, 1995). In a combined analysis of seven case-control studies with altogether 1122 patients with an invasive epithelial ovarian carcinoma and 5359 controls, the age-adjusted relative risk was estimated to be 5.4 (Hartge *et al.*, 1994). Familial aggregation of ovarian cancer, originally detected in individual families (Liber, 1950; Lewis and Clare Davison, 1969; Li *et al.*, 1970; Fraumeni *et al.*, 1975; Thor *et al.*, 1976), is thus convincingly demonstrated also in epidemiological studies.

Causes for this familial aggregation are largely unknown. An undetermined fraction of familial ovarian cancer is probably caused by inherited mutations in the recently cloned gene *BRCA1* (Miki *et al.*, 1994; Shattuck-Eidens *et al.*, 1995). Mutations in the *BRCA1* gene are estimated to be involved in 92% of breast-ovarian cancer families (Narod *et al.*, 1995), but site-specific ovarian cancer has also been reported to be linked to this gene (Steichen-Gersdorf *et al.*, 1994). Inherited mutations in the genes involved in the hereditary non-polyposis colorectal cancer, HNPCC, also predispose to ovarian cancer (Lynch *et al.*, 1986; Mecklin and Järvinen 1991; Aaltonen *et al.*, 1994). However, the most common form of familiality of ovarian cancer is the occurrence of only two ovarian cancer cases in the family, without features of these dominantly inherited cancer syndromes (Piver *et al.*, 1993; Greggi *et al.*, 1990; Grover *et al.*, 1993).

The present study aims to give a more precise picture of the familial risk of ovarian cancer than has been possible in previous studies. The Finns are a genetically homogeneous and stable population. Population registration in Finland is comprehensive and reliable. The nationwide Finnish Cancer Registry has been operating since 1953 registering over 99% of all solid tumors in Finland (Teppo *et al.*, 1994). It was possible therefore, for us to use high quality data from genealogical registers and medical records. With this population-based design we were able to avoid major biases in relation to patient selection, follow-up and risk estimation.

Correspondence: A Auranen
Received 1 December 1995; revised 1 February 1996; accepted 5 February 1996

### Materials and methods

### *Description of the data sources*

The population-based and nationwide Finnish Cancer Registry was founded in 1952 and cancer registration started in 1953. Reporting of cancer cases to the registry was made obligatory in 1961. Physicians, hospitals and pathology laboratories send reports to the registry independently. On average, five notifications are received per case. In the years 1953-1966, the registration was done manually using alphabetical patient name files. From the beginning of 1967 the register was computerised on the basis of the unique personal identification numbers given to residents of Finland. The Registry's files are annually linked to the file of deaths and emigrations issued by the Finland Statistics and Population Register Centre. Complete follow-up of cancer patients is achieved.

Population registration in Finland has traditions dating back to the 16th century and is considered to be of excellent quality. Local population registries are kept by the church parishes and, for people not belonging to any religious community, by local authorities. From 1964 this information is also registered in a nationwide population registry, kept by the Population Register Centre.

### *Index patients and relatives*

From the Finnish Cancer Registry, all women who had an invasive epithelial ovarian cancer diagnosed during the years 1980-1982 under 76 years of age, were selected as index patients; they numbered 863.

The local registries of the communities where the patients were born were contacted to obtain the names and birth dates of the parents and siblings. In cases in which the family had moved to another community, tracing was continued until either of the parents deceased or the mother reached 50 years of age and further pregnancies were considered unlikely. The parents and siblings were followed up through the parish records until death or until they obtained a personal identification number. The data on the children and husbands were obtained from the parishes or the Central Population Register. The data on relatives were linked with the Central Population Register in February 1994 to obtain dates of death of the relatives.

Tracing of family members was successful for 700 (81%) of the 863 index patients. Failure to trace the family members was due to: inability to find the patient in the population registries of her reported birthplace, probably owing to inaccurately provided birthplace (90 patients), lack of response from the local officials (25 patients), born abroad (five patients) or failure to follow the family until additional children were considered unlikely, caused by the fact that the family had changed location repeatedly (43 patients).

Among the above 700 families, follow-up for 273 persons altogether in 141 families had to be interrupted for practical causes before death or assignment of a personal identification number. In the remaining 559 families all the family members were followed up. The final analysis was restricted to these 559 families. The distribution of the 559 index patients according to age and histology is presented in Table I.

*Statistical methods*

The relatives were followed up for cancer through the files of the Finnish Cancer Registry. For relatives who died in the period 1953 to 1966 the follow-up was done manually using the alphabetical patient name files with date of birth and place of birth and residence as an additional key. For those alive after 1 January 1967 the follow-up was done automatically using the personal identification number as the key. For mothers and sisters deceased during the period 1936–52, death certificates were obtained to verify the cause of death. For people deceased before 1936, death certificates are not available.

To validate the data and to determine the starting point of the analysis, the overall cancer risk was first calculated separately for the period 1953–1966. The standardised incidence ratios (SIRs) for overall cancer in females and males were 0.7 (95% CI 0.5–1.0) and 0.7 (95% CI 0.5–1.0) respectively. This significant risk deficit was considered to reflect problems encountered in the manual follow-up of the relatives. In consequence, the data were not considered reliable for the period 1953–1966, and the main analysis was restricted to the period starting from 1 January 1967.

Follow-up for cancer among parents of the index patients started at date of birth of the index patient or on 1 January 1967, whichever was later, and ended at death or on 31 December 1993, whichever was first. For siblings and children the follow-up started at the date of their birth or on 1 January 1967, whichever was later. Siblings' person–years at risk during the follow-up were categorised into four age groups.

The numbers of observed cases and person–years at risk in each relative category were counted, by five year age groups, separately for three calendar periods (1967–75, 1976–84 and 1985–93). The expected numbers of cases for total cancer and for specific cancer types were calculated by multiplying the number of person–years in each age group by the corresponding period-specific cancer incidence in all of Finland.

The specific cancer types selected a priori for the analysis included the cancer sites with known or suspected exceptional risk in earlier studies, and other common cancer types to give the whole picture of the cancer situation among the cohort. The selected cancers were: ovary, breast, cervix uteri, endometrium, prostate, stomach, colon, rectum, lung, pancreas and melanoma of the skin.

Because of the definition of the index patients, the expected number for ovarian cancer during the period 1980–82 in ages below 75 was subtracted from the total expected number of cases for female overall cancer and ovarian cancer. The decision to exclude observed and expected numbers for the whole period 1980–82 is unbiased. There were no families with more than one ovarian cancer diagnosed in 1980–82.

Standardised incidence ratios (SIRs) were defined as ratios of the observed to the expected number of cases. The statistical significance was tested by the Mantel–Haenszel chi-square test and 95% confidence intervals (CIs) were calculated on the presumption that the number of observed cases followed a Poisson distribution.

### Results

In the 559 families there were 6501 first-degree relatives. Ovarian cancer was diagnosed in 31 relatives from 27 families. Eight of the cancers were diagnosed before the start of the follow-up period in 1967. Two ovarian cancers were present in 23 families (4.1% of all families) and three ovarian cancers in four families (0.7% of all families). Three mothers and 28 sisters were affected. In the four families with three ovarian cancer patients, only sisters were affected in two families and two sisters and a mother in one family. In the fourth family, only sisters were affected with ovarian cancer, but the mother had an abdominal cancer of undefined origin. Breast cancer was present in ten of these 27 families.

Of the relatives, 3072 were at risk on 1 January 1967 or thereafter. They were followed up for a total of 69 793 person–years (mean 23 years). The overall risk of cancer was not increased among the relatives (Table II). Female relatives had a significantly increased 2.8-fold risk (95% CI 1.8–4.2) for ovarian cancer, but no other significantly increased or decreased risks were observed (Table III). The mothers' SIR for ovarian cancer was 0.6 (0.0–3.1) while the sisters' SIR

**Table I** Distribution of 559 index patients diagnosed with invasive epithelial ovarian cancer under 76 years of age in Finland in 1980–82 by age and histology

| | |
|---|---|
| Age group | |
| <34 | 32 (6%) |
| 35–44 | 62 (11%) |
| 45–54 | 130 (23%) |
| 55–64 | 180 (32%) |
| 65–75 | 155 (28%) |
| Total | 559 (100%) |
| Histology | |
| Serous | 144 (26%) |
| Mucinous | 91 (16%) |
| Endometrioid | 50 (9%) |
| Clear cell | 24 (4%) |
| Anaplastic | 37 (7%) |
| Unspecified adenocarcinoma | 213 (38%) |
| Total | 559 (100%) |

**Table II** Numbers (*n*) of relatives and observed numbers (Obs) and standardised incidence ratios (SIR) with 95% confidence intervals (CI) of all cancers in the first-degree relatives in 1967–93 of 559 index patients diagnosed with an invasive epithelial ovarian cancer in Finland 1980–82

| *Relative* | n | *Obs* | *SIR* | *CI* |
|---|---|---|---|---|
| Female | 1605 | 156 | 1.0 | 0.8–1.1 |
| Mothers | 287 | 35 | 0.8 | 0.5–1.1 |
| Sisters | 918 | 116 | 1.0 | 0.9–1.2 |
| Daughters | 400 | 5 | 0.6 | 0.2–1.4 |
| Male | 1467 | 151 | 0.9 | 0.8–1.1 |
| Fathers | 171 | 39 | 1.1 | 0.8–1.4 |
| Brothers | 861 | 107 | 0.9 | 0.7–1.1 |
| Sons | 435 | 5 | 0.9 | 0.3–2.0 |

was 3.7 (2.3–5.7). The relative risk for ovarian cancer among sisters decreased by increasing age of the sister and by increasing age of the index patient (Table IV). Apart from ovarian cancer, no significantly increased or decreased risks were observed when the mothers and sisters were analysed separately. The SIR for breast cancer in mothers was 0.9 (0.4–1.8) and in sisters 1.0 (0.6–1.4).

In sisters, stratification of the data according to the age at diagnosis of the index patients into three groups (<45 years, 45–54 years and 55–75 years) revealed that the sisters of the youngest index patients had a significantly increased overall cancer risk (SIR 2.1, 1.1–3.6) and the risk was concentrated in sisters aged 45 to 59 years (SIR 2.9, CI 1.4–6.0). This excess was attributable to ovarian cancer only.

No significantly increased or decreased risks were observed in the male relatives (Table III). Division of the males into fathers and brothers did not change the results. For brothers, the data were further stratified according to the age of the index patient into three age groups, as described above. No increase in overall cancer risk or risk for any specific cancer was observed in any age category.

Of the 918 sisters at risk, 118 (13%) sisters belonged to families with breast cancer (either in index patient or relative). The risk of ovarian cancer was significantly higher among sisters from families with breast cancer (SIR 9.2, 3.7–19, $n=7$) compared with sisters from families with no breast cancer patients (SIR 2.9, 1.6–4.8, $n=15$, rate ratio 3.1, $P < 0.05$). In the 27 families with two or more ovarian cancers, 1.6 breast cancers were expected in the sisters but seven were observed (SIR 4.3, 1.7–8.8). The risk was significantly increased only in sisters between 60 and 74 years of age (SIR 6.7, CI 1.8–17.1). The occurrence of breast cancer or more than one ovarian cancer in the family did not affect the brothers' risk for cancer in any of the analysed sites, and the occurrence of gastric or colorectal cancer in the families did not increase the brothers' or the sisters' risk for any cancers.

**Table III** Observed (Obs) and expected (Exp) numbers and standardised incidence ratios (SIR) with 95% confidence intervals (CI) of specific cancers in the first-degree relatives in 1967–93 of 559 index patients diagnosed with an invasive ovarian cancer in Finland in 1980–82

| *Site* | *Obs* | *Exp* | *SIR* | *95% CI* |
|---|---|---|---|---|
| Breast | 37 | 38.8 | 0.9 | 0.7–1.3 |
| Ovary | 23 | 8.2 | 2.8 | 1.8–4.2 |
| Cervix uteri | 6 | 5.0 | 1.2 | 0.4–2.6 |
| Endometrium | 14 | 10.1 | 1.4 | 0.8–2.3 |
| Prostate | 18 | 23.3 | 0.8 | 0.5–1.2 |
| Stomach | | | | |
| Female | 11 | 12.9 | 0.8 | 0.4–1.5 |
| Male | 20 | 15.4 | 1.3 | 0.8–2.0 |
| Colon | | | | |
| Female | 6 | 10.3 | 0.6 | 0.2–1.3 |
| Male | 6 | 6.8 | 0.9 | 0.3–1.9 |
| Rectum | | | | |
| Female | 6 | 6.7 | 0.9 | 0.3–2.0 |
| Male | 6 | 6.3 | 1.0 | 0.4–2.1 |
| Pancreas | | | | |
| Female | 4 | 6.9 | 0.6 | 0.2–1.5 |
| Male | 6 | 6.3 | 1.0 | 0.4–2.1 |
| Lung | | | | |
| Female | 7 | 6.6 | 1.1 | 0.4–2.2 |
| Male | 40 | 44.0 | 0.9 | 0.6–1.2 |
| Melanoma | | | | |
| Female | 1 | 3.6 | 0.3 | 0.0–1.6 |
| Male | 4 | 3.2 | 1.3 | 0.4–3.2 |

### Discussion

In the present study of Finnish patients we were able to demonstrate familial aggregation of ovarian cancer in 27 of 559 studied families. The relative risk for ovarian cancer among first-degree relatives was 2.8 being basically of similar magnitude to that reported in previous studies (Parazzini *et al.*, 1992; Houlston *et al.*, 1993; Hartge *et al.*, 1989; Schildkraut *et al.*, 1989; Kerber and Slattery 1995). The cumulative incidence of ovarian cancer in Finland to the age of 75 is 1.4%. Presuming a minimum life-time relative risk of 3.5 in sisters, this corresponds to a minimum 5% likelihood of getting ovarian cancer by this age.

The significance of the age at onset of ovarian cancer to the familiality of the disease has been a controversial issue. Some studies have detected no relationship between familiality and age at onset of ovarian cancer (Parazzini *et al.*, 1992; Narod *et al.*, 1994; Kerber and Slattery 1995), while other studies suggest that familial occurrence of ovarian cancer is increased at younger (Lynch *et al.*, 1993; Houlston *et al.*, 1993) or older (Schildkraut *et al.*, 1989) ages at onset.

The relation of young age to the familiality of the disease, observed in this study, is typical for inherited predisposition and resembles the pattern previously observed (Houlston *et al.*, 1993). Their data were collected from healthy relatives consulting an ovarian cancer screening clinic. Since relatives of younger cancer patients could be presumed to be more worried about the familiality of cancer than relatives of older patients, a possible bias towards younger index patients might have occurred in their study. Such a bias does not exist in our population-based study.

The observation that only sisters had an increased risk for ovarian cancer, whereas no increase in risk could be observed for mothers, would speak for a recessive rather than a dominant mode of transmission. However, close to 50% of mothers were deceased before the follow-up period. During the follow-up period starting from 1967, the number of person–years at risk for mothers at younger ages is limited and the age patterns of person–years at risk are different for mothers and sisters. The overall difference between the SIRs of the sisters and mothers (higher SIR among sisters than mothers) is not statistically significant, and because of the decreasing relative risk by increasing age, adjustment for age would further diminish this difference.

When the causes of death for relatives who died before 1967 were verified from death certificates or from the Finnish Cancer Registry, only two further ovarian cancers were found among the mothers. The notable lack of ovarian cancer in the mothers may implicate recessive inheritance or shared environmental risk factors in sisters. Although the findings

**Table IV** Observed (Obs) numbers and standardised incidence ratios (SIR) with 95% confidence intervals (CI) of ovarian cancer by age of the index patient and by age of the sister in the sisters of the 559 index patients diagnosed with invasive epithelial ovarian cancer in Finland 1980–82

| | *Age of the index patient at diagnosis* | | | | | | | | | | | |
|---|---|---|---|---|---|---|---|---|---|---|---|---|
| *Age of the sister* | <45 years | | | *45–54 years* | | | *55–75 years* | | | *Whole cohort* | | |
| *during follow-up* | *Obs* | *SIR* | *95% CI* | *Obs* | *SIR* | *95% CI* | *Obs* | *SIR* | *95% CI* | *Obs* | *SIR* | *95% CI* |
| 30–44 | 2 | 15.4 | 1.9–55.6 | 1 | 5.6 | 0.1–30.9 | 1 | 8.3 | 0.2–46.4 | 4 | 9.3 | 2.5–23.8 |
| 45–59 | 1 | 5.6 | 0.1–31.0 | 4 | 5.9 | 1.6–15.1 | 3 | 2.5 | 0.5–7.2 | 8 | 3.9 | 1.7–7.6 |
| 60–74 | – | 0.0 | 0.0–63.2 | 1 | 2.3 | 0.0–13.0 | 9 | 4.3 | 2.0–8.1 | 10 | 3.8 | 1.8–7.1 |
| 75+ | – | 0.0 | 0.0–4100 | – | 0.0 | 0.0–189 | – | 0.0 | 0.0–5.2 | – | 0.0 | 0.0–5.0 |
| All ages | 3 | 7.3 | 1.5–21.4 | 6 | 4.5 | 1.6–9.8 | 13 | 3.1 | 1.7–5.4 | 22 | 3.7 | 2.3–5.7 |

in most studies speak for a dominantly inherited predisposition (Houlston et al., 1991; Lynch et al., 1991), the possibility of a recessive inheritance, based on the observation of consanguinity among patients with ovarian cancer, has also been raised (Cramer et al., 1983). In the OPCS study from England and Wales (Easton et al., 1996), sisters of ovarian cancer cases had higher mortality from this disease than mothers, which supports the observations in the present study.

We did not detect an increase in breast cancer risk among all the relatives of the ovarian cancer patients. Ovarian cancer is known to be genetically related to breast cancer in the breast–ovarian cancer syndrome (Lynch et al., 1974; Go et al., 1983), which is mainly caused by inherited mutations in the recently cloned *BRCA1* gene (Miki et al., 1994; Narod et al., 1995). Some of the previous studies have detected a small but significant increase in breast cancer risk among relatives of ovarian cancer patients, which has been interpreted as reflecting the existence of the breast–ovarian cancer syndrome in the study populations (Houlston et al., 1993; Schildkraut et al., 1989). If so, our finding might imply that *BRCA1* gene mutations causing both breast and ovarian cancer are so infrequent among Finnish ovarian cancer patients that their effect is not visible at the population level.

The association of breast and ovarian cancer was also evident in the present study. In all, 30% of ovarian cancers in sisters were observed among the 13% of sisters belonging to families with breast cancer. However, 70% of familial ovarian cancer could not be explained by coaggregation of breast and ovarian cancer, which suggests that in the majority of the families with two or more ovarian cancers, inherited mutations predisposing to both breast and ovarian cancer are not involved. In the OPCS study (Easton et al., 1996), it was calculated on the basis of *BRCA1* gene frequency estimates that *BRCA1* would account for 57% of the excess familial risk of ovarian cancer below age 70.

In contrast to previous studies (Cramer et al., 1983; Schildkraut et al., 1989; Slattery and Kerber 1994) we did not detect any increased risk of colon or gastric cancer in the relatives of ovarian cancer patients. Ovarian cancer and colon cancer are known to be genetically related in the hereditary non-polyposis colorectal cancer (HNPCC) syndrome (Lynch et al., 1986; Aaltonen et al., 1994), but ovarian cancer is rare in this syndrome, representing only 4% of the cancers in the HNPCC families (Mecklin and Järvinen 1991). Typical HNPCC families are rare in Finland: less than a hundred typical HNPCC families have been identified to date (L Aaltonen, personal communication). We presume that no HNPCC families were among the studied families.

Only index patients having an invasive cancer with histology coded as epithelial were selected for this study. Close to 40% of these cancers were reported to the Cancer Registry only as adenocarcinoma without further specification. Therefore, analysis of separate histologies was not considered useful. The significance of histology can be better evaluated by investigating the familial ovarian tumours in more detail.

The strength of this study was the reliable detection of the relatives' cancers through the nationwide Finnish Cancer Registry. It was also possible for us to compare the cancer risk among the relatives with that in the underlying population drawn from the same database as the observed cases; potential biases involving differences between numerator and denominator were thereby avoided. Using incidence instead of mortality avoids errors in the stated underlying cause of death and also the biases caused by differences in survival of cases in the cohort and referent population.

This dataset represents all the familial cases of ovarian cancer that could be detected among Finnish ovarian cancer patients diagnosed during a 3 year period, regardless of the cause of the familiality. The sisters of ovarian cancer patients were found to have a significantly increased risk for ovarian cancer, and the risk increase was age related, being highest in the sisters of the youngest ovarian cancer patients. Nothing conclusive can be stated about the mode of inheritance, but the possibility of recessive inheritance was not excluded. Less than half of this observed familiality could be explained by coaggregation of breast and ovarian cancer in the family. A detailed genetic analysis of the tumours from these patients with familial ovarian cancer should further clarify whether the observed familiality is partly or completely caused by already known gene mutations or whether other as yet unknown mechanisms or gene defects are also involved.

**Acknowledgements**
This study was supported by the Finnish Cancer Foundation, the Turku University Foundation and the Ida Montin Foundation.

### References

AALTONEN LA, PELTOMÄKI P, MECKLIN J-P, JÄRVINEN H, JASS JR, GREEN JS, LYNCH HT, WATSON P, TALLQVIST G, JUHOLA M, SISTONEN P, HAMILTON SR, KINZLER KW, VOGELSTEIN B AND DE LA CHAPELLE A. (1994). Replication errors in benign and malignant tumors from hereditary nonpolyposis colorectal cancer patients. *Cancer Res.*, **54**, 1645–1648.

CRAMER DW, HUTCHISON GB, WELCH WR, SCULLY RE AND RYAN KJ. (1983) Determinants of ovarian cancer risk. I. Reproductive experiences and family history. *J. Natl Cancer Inst.*, **71**, 711–716.

EASTON DF, MATTHEWS FE, FORD D, SWERDLOW AJ AND PETO J. (1996). Cancer mortality in relatives of women with ovarian cancer: the OPCS study. *Int. J. Cancer*, **65**, 284–294.

FRAUMENI JF, GRUNDY GW, CREAGAN ET AND EVERSON RB. (1975). Six families prone to ovarian cancer. *Cancer*, **36**, 364–369.

GO RC, KING MC, BAILEY WJ, ELSTON RC, AND LYNCH HT. (1983). Genetic epidemiology of breast cancer and associated cancers in high risk families: I. segregation analysis. *J. Natl Cancer Inst.*, **71**, 455–461.

GREGGI S, GENUARDI M, BENEDETTI-PANICI P, CENTO R, SCAMBIA G, NERI G AND MANCUSO S. (1990). Analysis of 138 consecutive ovarian cancer patients: incidence and characteristics of familial cases. *Gynecol. Oncol.*, **39**, 300–304.

GROVER S, QUINN MA AND WEIDEMAN P. (1993). Patterns of inheritance of ovarian cancer. An analysis from an ovarian cancer screening program. *Cancer*, **72**, 526–530.

HARTGE P, SCHIFFMAN MH, HOOVER R, McGOWAN L, LESHER L AND NORRIS HJ. (1989). A case–control study of epithelial ovarian cancer. *Am. J. Obstet. Gynecol.*, **161**, 10–16.

HARTGE P, WHITTEMORE AS, ITNYRE J, McGOWAN L, CRAMER D AND THE COLLABORATIVE OVARIAN CANCER GROUP. (1994). Rates and risks of ovarian cancer in subgroups of white women in the United States. *Obstet. Gynecol.*, **84**, 760–764.

HOULSTON RS, COLLINS A, SLACK J, CAMPBELL S, COLLINS WP, WHITEHEAD MI AND MORTON NE. (1991). Genetic epidemiology of ovarian cancer: segregation analysis. *Ann. Hum. Genet.*, **55**, 291–299.

HOULSTON RS, BOURNE TH, COLLINS WP, WHITEHEAD MI, CAMPBELL S AND SLACK J. (1993). Risk of ovarian cancer and genetic relationship to other cancers in families. *Hum. Hered.*, **43**, 111–115.

KERBER RA AND SLATTERY ML. (1995). The impact of family history on ovarian cancer risk. The Utah population database. *Arch. Intern. Med.*, **155**, 905–912.

LEWIS ACW AND CLARE DAVISON BC. (1969). Familial ovarian cancer. *Lancet*, **2**, 235–237.

LI FP, RAPOPORT AH, FRAUMENI JF AND JENSEN RD. (1970). Familial ovarian carcinoma. *J. Am. Med. Ass.*, **214**, 1559–1561.

LIBER AF. (1950). Ovarian cancer in mother and five daughters. *Arch. Pathol.*, **49**, 280–290.

LYNCH HT, GUIRGIS HA, ALBERT S, BRENNAN M, LYNCH J, KRAFT C, POCEKAY D, VAUGHNS C AND KAPLAN A. (1974). Familial association of carcinoma of the breast and ovary. *Surg. Gynecol. Obst.*, **138**, 717–724.

LYNCH HT, BEWTRA C AND LYNCH JF. (1986). Familial ovarian carcinoma. Clinical nuances. *Am. J. Med.*, **81**, 1073–1076.

LYNCH HT, CONWAY T AND LYNCH J. (1991). Hereditary ovarian cancer. Pedigree studies, part II. *Cancer Genet. Cytogenet.*, **52**, 161–183.

LYNCH HT, WATSON P, LYNCH JF, CONWAY TA AND FILI M. (1993). Hereditary ovarian cancer. Heterogeneity in age at onset. *Cancer*, **71**, 573–581.

MECKLIN J-P AND JÄRVINEN HJ. (1991). Tumor spectrum in Cancer Family Syndrome (hereditary nonpolyposis colorectal cancer). *Cancer*, **68**, 1109–1112.

MIKI Y, SWENSEN J, SHATTUCK-EIDENS D, FUTREAL PA, HARSHMAN K, TAVTIGIAN S, LIU Q, COCHRAN C, BENNETT LM, DING W, BELL R, ROSENTHAL J, HUSSEY C, TRAN T, MCCLURE M, FRYE C, HATTIER T, PHELPS R, HAUGEN-STRANO A, KATCHER H, YAKUMO K, GHOLAMI Z, SHAFFER D, STONE S, BAYER S, WRAY C, BOGDEN R, DAYANANTH P, WARD J, TONIN P, NAROD S, BRISTOW PK, NORRIS FH, HELVERING L, MORRISON P, ROSTECK P, LAI M, BARRETT JC, LEWIS C, NEUHAUSEN S, CANNON-ALBRIGHT L, GOLDGAR D, WISEMAN R, KAMB A AND SKOLNICK MH. (1994). A strong candidate for the breast and ovarian cancer susceptibility gene *BRCA1*. *Science*, **266**, 66–71.

NAROD SA, MADLENSKY L, BRADLEY L, COLE D, TONIN P, ROSEN B AND RISCH HA. (1994). Hereditary and familial ovarian cancer in southern Ontario. *Cancer*, **74**, 2341–2346.

NAROD SA, FORD D, DEVILEE P, BARKARDOTTIR RB, LYNCH HT, SMITH SA, PONDER BAJ, WEBER BL, GARBER JE, BIRCH JM, CORNELIS RS, KELSELL DP, SPURR NK, SMYTH E, HAITES N, SOBOL H, BIGNON Y-J, CHANG-CLAUDE J, HAMANN U, LINDBLOM A, BORG A, PIVER MS, GALLION HH, STRUEWING JP, WHITTEMORE A, TONIN P, GOLDGAR DE, EASTON DF AND THE BREAST CANCER LINKAGE CONSORTIUM. (1995). An evaluation of genetic heterogeneity on 145 breast–ovarian cancer families. *Am. J. Hum. Genet.*, **56**, 254–264.

PARAZZINI F, NEGRI E, LA VECCHIA C, RESTELLI C AND FRANCESCHI S. (1992). Family history of reproductive cancers and ovarian cancer risk: an Italian case–control study. *Am. J. Epidemiol.*, **135**, 35–40.

PIVER MS, BAKER TR, JISHI MF, SANDECKI AM, TSUKADA Y, NATARAJAN N, METTLIN CJ AND BLAKE CA. (1993). Familial ovarian cancer. A report of 658 families from the Gilda Radner familial ovarian cancer registry 1981–1991. *Cancer*, **71**, 582–588.

SCHILDKRAUT JM, RISCH N AND THOMPSON D. (1989). Evaluating genetic association among ovarian, breast and endometrial cancer: evidence for a breast/ovarian cancer relationship. *Am. J. Hum. Genet.*, **45**, 521–529.

SHATTUCK-EIDENS D, MCCLURE M, SIMARD J, LABRIE F, NAROD S, COUCH F, HOSKINS K, WEBER B, CASTILLA L, ERDOS M, BRODY L, FRIEDMAN L, OSTERMEYER E, SZABO C, KING M-C, JHANWAR S, OFFIT K, NORTON L, GILEWSKI T, LUBIN M, OSBORNE M, BLACK D, BOYD M, STEEL M, INGLES S, HAILE R, LINDBLOM A, OLSSON H, BORG A, BISHOP DT, SOLOMON E, RADICE P, SPATTISTA G, GAYTHER S, PONDER B, WARREN W, STRATTON M, LIU Q, FUJIMARA F, LEWIS C, SKOLNICK MH AND GOLDGAR DE. (1995). A collaborative survey of 80 mutations in the *BRCA1* breast and ovarian cancer susceptibility gene. *J. Am. Med. Ass.*, **273**, 535–541.

SLATTERY ML AND KERBER RA. (1994). Family history of cancer and colon cancer risk: the Utah population database. *J. Natl Cancer Inst.*, **86**, 1618–1626.

STEICHEN-GERSDORF E, GALLION HH, FORD D, GIRODET C, EASTON DF, DICIOCCIO RA, EVANS G, PONDER MA, PYE C, MAZOYER S, NOGUCHI T, KARENGUEVEN F, SOBOL H, HARDOUIN A, BIGNON Y-J, PIVER MS, SMITH SA AND PONDER BAJ. (1994). Familial site-specific ovarian cancer is linked to *BRCA1* on 17q12–21. *Am. J. Hum. Genet.*, **55**, 870–875.

TEPPO L, PUKKALA E AND LEHTONEN M. (1994). Data quality and quality control of a population-based cancer registry. Experience in Finland. *Acta Oncol.*, **33**, 365–369.

THOR L, PERSSON BH AND KJESSLER B. (1976). Familial ovarian carcinoma. *Uppsala J. Med. Sci.*, **81**, 189–191.